# Fabrication of single color centers in sub-50 nm nanodiamonds using ion implantation

**DOI:** 10.1515/nanoph-2022-0678

**Published:** 2023-02-01

**Authors:** Xiaohui Xu, Zachariah O. Martin, Michael Titze, Yongqiang Wang, Demid Sychev, Jacob Henshaw, Alexei S. Lagutchev, Han Htoon, Edward S. Bielejec, Simeon I. Bogdanov, Vladimir M. Shalaev, Alexandra Boltasseva

**Affiliations:** Elmore Family School of Electrical and Computer Engineering, Birck Nanotechnology Center, Purdue University, West Lafayette, IN 47907, USA; Elmore Family School of Electrical and Computer Engineering, Birck Nanotechnology Center, Purdue Quantum Science and Engineering Institute (PQSEI), Purdue University, West Lafayette, IN 47907, USA; The Quantum Science Center (QSC), a National Quantum Information Science Research Center of the U.S. Department of Energy (DOE), Oak Ridge National Laboratory, Oak Ridge, TN 37831, USA; Sandia National Laboratories, Albuquerque, NM 87123, USA; Los Alamos National Laboratory, Los Alamos, NM 87545, USA; Department of Electrical and Computer Engineering, Nick Holonyak, Jr. Micro and Nanotechnology Laboratory, Illinois Quantum Information Science and Technology Center, University of Illinois at Urbana-Champaign, Urbana, IL 60801, USA

**Keywords:** color centers, ion implantation, nanodiamond, quantum nanophotonics, single-photon emitters

## Abstract

Diamond color centers have been widely studied in the field of quantum optics. The negatively charged silicon vacancy (SiV^−^) center exhibits a narrow emission linewidth at the wavelength of 738 nm, a high Debye–Waller factor, and unique spin properties, making it a promising emitter for quantum information technologies, biological imaging, and sensing. In particular, nanodiamond (ND)-based SiV^−^ centers can be heterogeneously integrated with plasmonic and photonic nanostructures and serve as *in vivo* biomarkers and intracellular thermometers. Out of all methods to produce NDs with SiV^−^ centers, ion implantation offers the unique potential to create controllable numbers of color centers in preselected individual NDs. However, the formation of single color centers in NDs with this technique has not been realized. We report the creation of single SiV^−^ centers featuring stable high-purity single-photon emission through Si implantation into NDs with an average size of ∼20 nm. We observe room temperature emission, with zero-phonon line wavelengths in the range of 730–800 nm and linewidths below 10 nm. Our results offer new opportunities for the controlled production of group-IV diamond color centers with applications in quantum photonics, sensing, and biomedicine.

## 1 Introduction

The development of bright, stable quantum emitters (QEs) is critical for the advancement of multiple areas across bioscience and quantum photonic technologies, including photon-based biosensing, super-resolution imaging, quantum computation, and secure quantum communication [1–7]. Over the past years, a plethora of QEs have been demonstrated in various material systems [8–10]. The negatively charged silicon vacancy (SiV^−^) center in diamond has received significant interest due to its narrow zero-phonon line (ZPL), a high Debye–Waller factor (>0.7), weak spectral diffusion owing to atomic inversion symmetry, and robust optical transitions [11–15]. The SiV^−^ center also has an electronic spin that can be optically initiated, read out [16], and coherently controlled by microwaves [17], making it a promising spin–photon interface for quantum memories [18, 19]. In addition, the large strain susceptibility of SiV^−^ opens up opportunities for phonon-mediated hybrid quantum systems [20, 21].

Single SiV^−^ centers have been reported in both bulk diamond and in nanodiamonds (NDs). Compared to bulk diamond, NDs containing SiV^−^ centers possess some attractive properties. Using the recently developed pick-and-place technique [22, 23] based on atomic force microscopy, individually selected SiV-NDs can be deterministically transferred and integrated with photonic and/or plasmonic structures [24, 25] (e.g., photonic crystals, microcavities, nanoantennas) on various photonic platforms. The small dimensions of NDs enable effective coupling of color centers with photonic modes, which can be challenging for color centers in bulk diamond. SiV-NDs emitting in the near-infrared range can be safely embedded into biological tissues/cells for bioimaging, drug delivery, or other biomedical applications [7, 26, 27]. Furthermore, the phonon density of states in NDs could be altered compared to bulk diamond [28], hence reducing phonon-induced decoherence processes [29] and prolonging the coherence times of SiV^−^ centers.

There are three common methods for fabricating ND-based SiV^−^ centers. In the first method, NDs are chemically synthesized with the introduction of Si atoms, such as in the chemical vapor deposition (CVD) [30] and the high pressure, high temperature (HPHT) approach [31, 32]. In the second method, NDs are mechanically milled from bulk diamond already containing SiV^−^ centers (e.g., bead-assisted sonic disintegration (BASD) [33]). The third method involves NDs formed through detonation with Si-containing dopants [34].

Recently, SiV^−^ centers have been reported to form through Si ion implantation into NDs. Compared to methods mentioned above, ion implantation offers the possibility to control the number and positions of quantum emitters created [35, 36]. While it has been widely used to introduce various types of single color centers in bulk diamond [37–41], ion implantation into NDs poses several challenges. The nanoscale implantation cross section and higher susceptibility to ion beam damage [42] make it difficult to optimize ion irradiation conditions and significantly reduce the yield of single-photon emitter (SPE) generation in NDs. The fabrication of SiV^−^ centers in NDs with ion implantation has been demonstrated by several groups [43–46]. However, none of them reported high-purity antibunched light emission with the second-order correlation function *g*^2^(*t* = 0) < 0.5, indicating that SiV^−^ emitters are created in clusters rather than isolated single defects. Remarkably, Takashima et al. [46] observed photon antibunching of about *g*^(2)^(0) = 0.5 from SiV^−^NDs corresponding to as few as 2 single defects. In this work, we created single color centers with high single-photon purity in NDs with an average size of ∼20 nm using the ion implantation technique. We report the photophysics of the resulting color centers and investigate the inhomogeneous distribution of emission wavelengths. Finally, we discuss the impact and potential applications of the implantation method.

## 2 Experimental results

**Figure 1:**
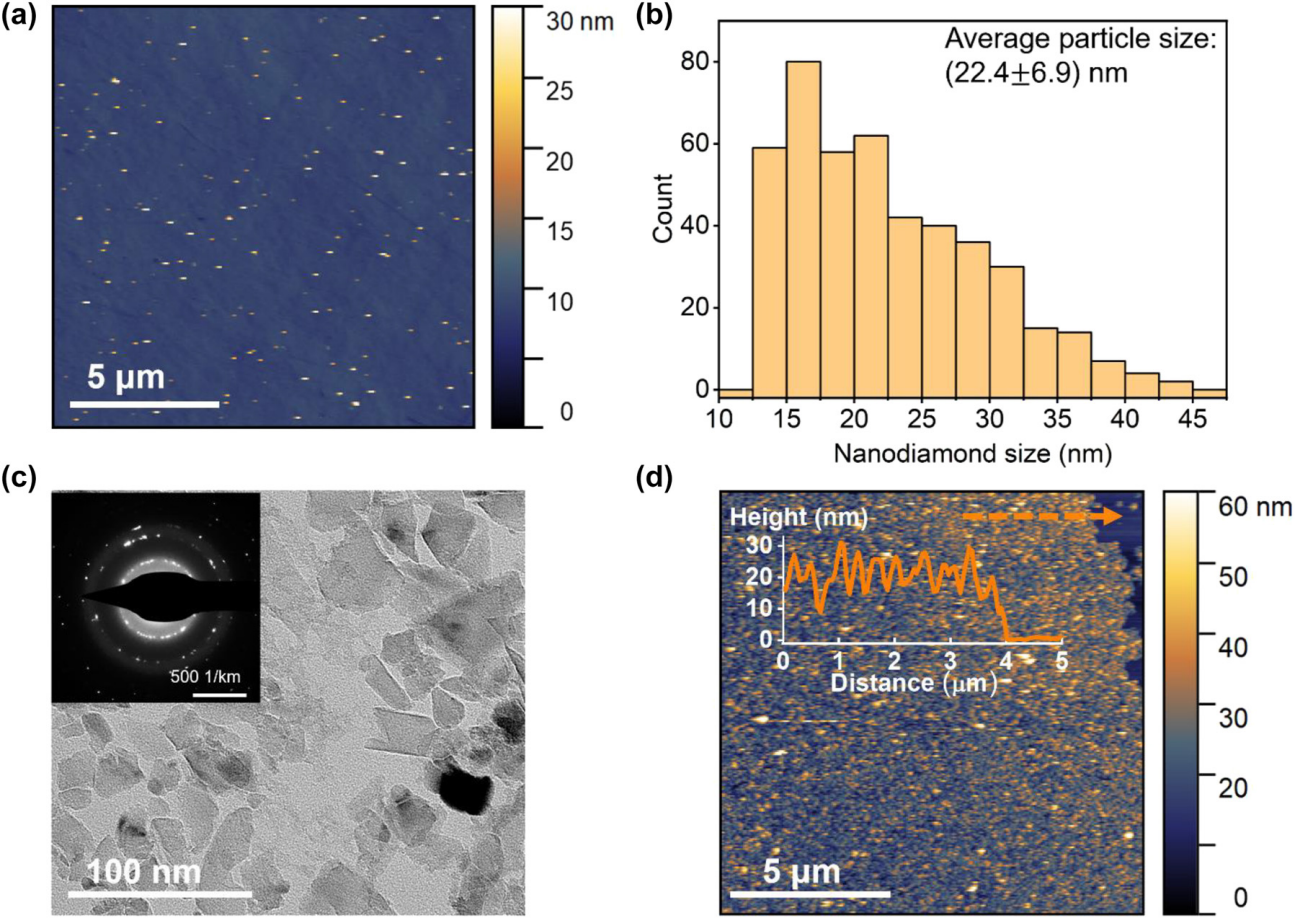
Characterization of non-fluorescent NDs prior to ion implantation. (a) AFM image of nanodiamonds drop casted from a suspension of 4 μg/mL onto a quartz coverslip. (b) Histogram showing the ND size distribution. Sizes are collected from 449 isolated NDs in AFM scans. Particles with heights <12 nm are excluded from the statistics as they could be dirt or features on the substrates. (c) Bright-field TEM image of a cluster of NDs. Inset: electron diffraction pattern taken over the entire area. (d) AFM image of NDs on a quartz coverslip for ion implantation experiments. The sample is drop casted from a suspension of 400 μg/mL. Inset: a height profile along the dashed orange line, illustrating a ND layer thickness of ∼20 nm that is comparable to the average height/size of NDs.

Prior to implantation, we statistically sized the nonfluorescent NDs obtained from Adamas Nanotechnologies. Figure 1a shows an atomic force microscope (AFM) image of individual diamond particles uniformly dispersed on a quartz coverslip substrate, without notable aggregation. The size distribution of NDs obtained from the AFM measured heights of over 400 individual particles is shown on Figure 1b. The investigated NDs featured an average height of 22.4 ± 6.9 nm with no particles larger than 50 nm. A bright field TEM image (Figure 1c) of an ND cluster shows NDs with irregular shapes and dimensions in the range of 10–50 nm, in agreement with the AFM results. A diffraction pattern registered over a cluster reveals randomly distributed diffraction spots due to the distinct orientations of diamond crystal planes in these nanoparticles. This pattern confirms the crystalline nature of our NDs. For ion implantation, we drop casted a high-density ND solution onto quartz coverslips, forming a nearly continuous layer of NDs (Figure 1d).

**Figure 2:**
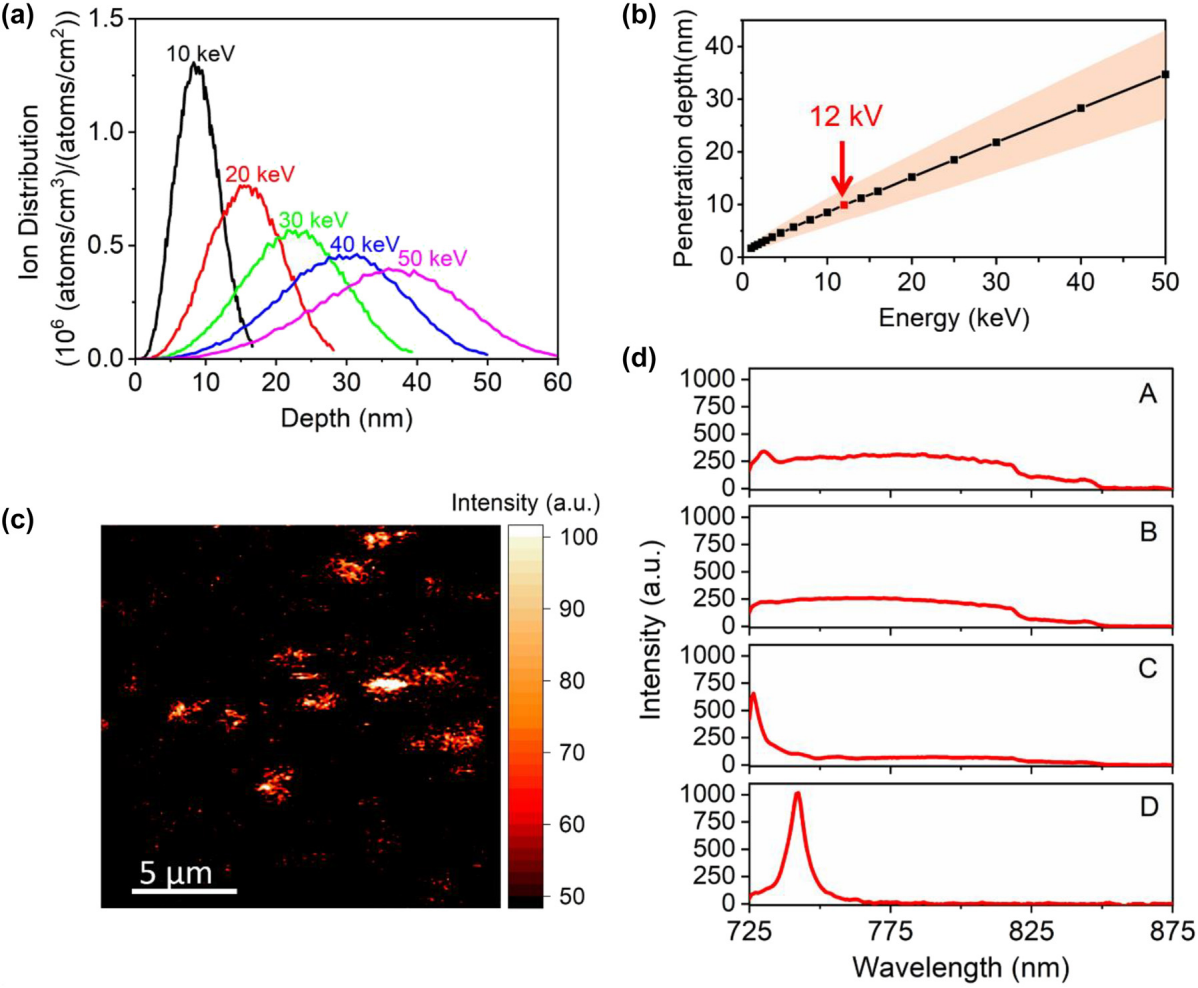
Optimization of the implantation parameters. (a) The implantation depth distribution of Si ions into diamond under different ion implantation energies, obtained from SRIM simulations. (b) The average penetration depth of Si ions as a function of ion implantation energy obtained from SRIM simulations. The yellow shaded area represents standard deviation from average values. The red data point indicates the ion energy (12 keV) used for implantation in this work. (c) A typical photoluminescence (PL) scan on NDs after ion implantation and postannealing, with isolated fluorescing spots. (d) Typical emission spectra of fluorescent spots on ND samples that are implanted with ion fluences of 2 × 10^10^, 1 × 10^12^, 5 × 10^13^, and 1 × 10^15^ ions/cm^2^ after annealing, noted as sample A, B, C, and D, respectively (as indicated in the graph). All spectra are collected under a 690 nm excitation laser at ∼2.6 mW, with the same integration time of 60 s. A long pass filter is used to cut out any fluorescence with wavelengths shorter than 725 nm. This results in the sharp edge formed at ∼725 nm in the spectra of sample C. Only the sample implanted at 1 × 10^15^ ions/cm^2^ shows a sharp emission peak centered around ∼740 nm, indicative of SiV emission.

The optimal energy of Si ions for implantation into NDs was determined using the Stopping and Range of Ions in Matter (SRIM) simulation [47]. Figure 2a shows the simulated distribution of Si ion penetration depth at ion energies ranging from 10 keV to 50 keV. The average penetration depth of ions increases nearly linearly with the ion energy (Figure 2b). The depth uncertainty (straggling) increases too, due to a higher probability of ion scattering in the diamond lattice. Considering that most NDs have sizes of 15–30 nm along the ion penetration direction, we choose the ion implantation energy of 12 keV, which corresponds to an average ion penetration depth of 10 ± 3 nm. To investigate the impact of implantation fluence at room temperature, four different fluences, i.e., 2 × 10^10^, 1 × 10^12^, 5 × 10^13^, and 1 × 10^15^ ions/cm^2^, were tested. Following the ion implantation procedure, NDs are annealed at high temperatures for color center activation and amorphous carbon removal (Materials and methods section). AFM measurements are also performed after annealing, which confirmed the neglectable effect of implantation/annealing on the size of NDs (Supplementary Materials). The samples are then characterized using a confocal microscope. Under the excitation of a 690 nm continuous-wave (CW) laser, photoluminescence (PL) maps scanned from ND clusters show bright spots on top of a low-fluorescence background (Figure 2c). Typical PL emission spectra of isolated bright spots collected from the four samples are illustrated in Figure 2d. Sample A, B, and C emit over a broad spectral range without any pronounced peaks in the transmission range of our 725 nm lowpass pump rejection filter (Figure 2d). When the ion fluence increases to 1 × 10^15^ ions/cm^2^, sharp spectral PL peaks are observed, with an example shown in Figure 2d. A sharp peak centered at ∼740 nm roughly matches the most commonly reported ZPL wavelength of SiV^−^ centers in NDs [45, 48]. This indicates the successful creation of SiV^−^ defects in sub-50 nm NDs.

**Figure 3:**
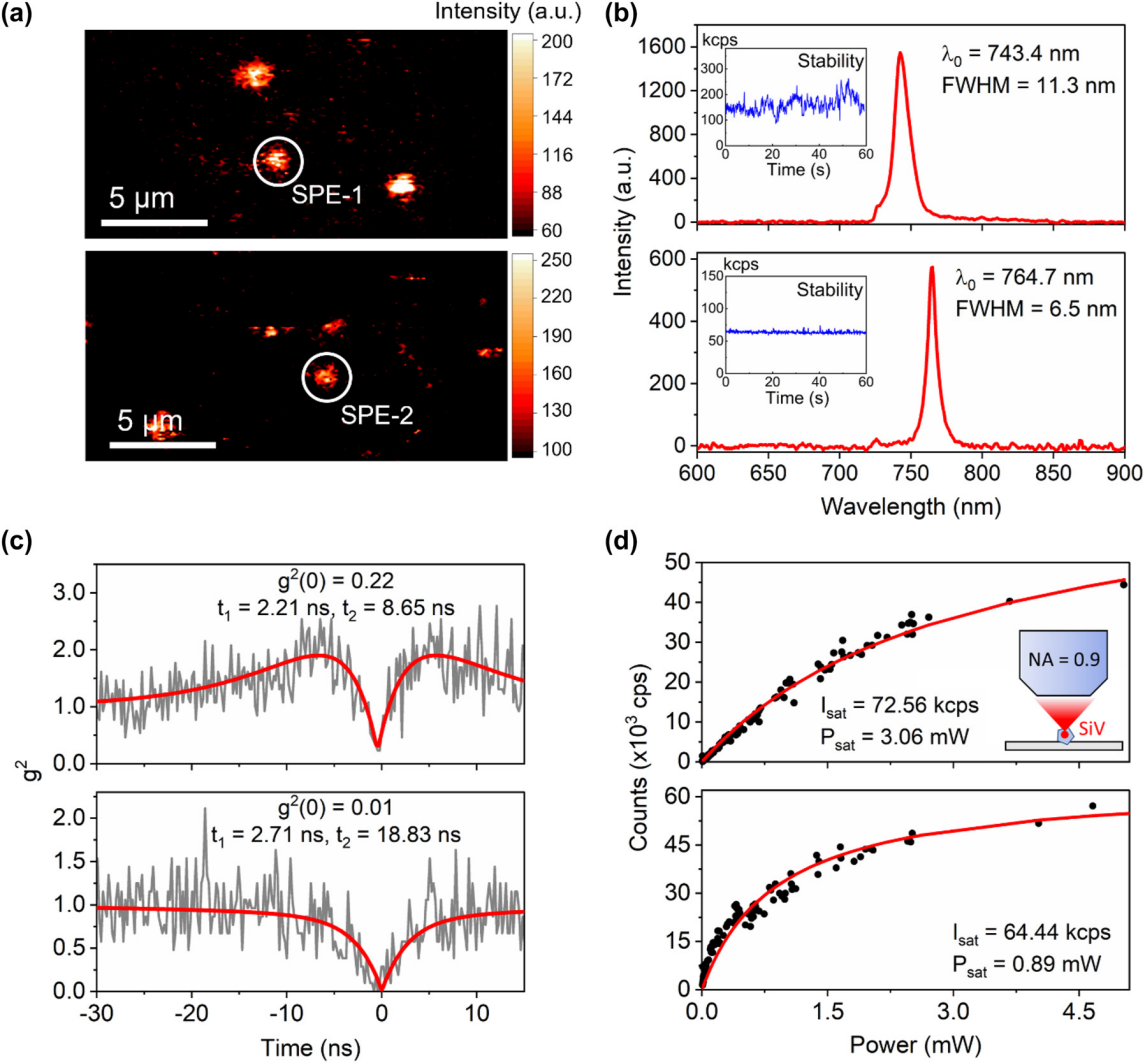
Photophysics of typical single-photon emitters (SPEs) found on sample D. (a) Two PL maps containing bright, stable emitters collected on sample D under a 690 nm CW laser excitation. (b) The emission spectra of emitter SPE-1 (top) and SPE-2 (bottom) circled in (a) under the excitation of the 690 nm CW laser. Insets are the fluorescence stability curves of both emitters recorded for 60 s. (c) Second-order autocorrelation measurement of SPE-1 (top) and SPE-2 (bottom). Gray lines are experimental data and red lines are fitted curves using the three-level model mentioned in the main text. The graph indicates the fitted values of *g*^(2)^(0), *t*_1_, and *t*_2_. (d) Fluorescence saturation curves of typical SPEs found on sample D, showing saturation counts of around 70 kcps. Solid black dots are experimental data and red lines are fitted curves. Inset: a schematic illustrating the collection configurations used for SiV centers in NDs on a quartz coverslip.

Next, we focus on sample D to investigate the optical properties of the created SiV^−^ centers. PL maps over a sample area of ∼4000 µm^2^ were collected, and isolated emission spots were characterized spectrally and temporally. Figure 3a shows example PL maps from two scanned areas containing stable emitters. Two emitters noted as SPE-1 and SPE-2 are identified through sharp emission peaks centered at 743.4 nm and 764.7 nm, respectively. The emission’s full widths at half-maxima (FWHM) are estimated to be 11.3 nm and 6.5 nm (Figure 3b), by fitting the peaks with a Lorentzian function. Note that the emission peak position of SPE-2 is significantly shifted from the typical ZPL of SiV^−^ emitters at room temperature, which we will discuss in more detail below. Both emitters exhibit stable photoluminescence emission during the optical characterization without notable blinking or bleaching. The second-order autocorrelation (*g*^(2)^(*t*)) data of emitters can be fitted using a three-level model [13]: *g*^(2)^(*t*) = 1 − *α*·exp(−|*t*|/*t*_1_) + *β*·exp(−|*t*|/*t*_2_), from which the autocorrelation at zero time delay *g*^(2)^(0) can be fitted as *g*^(2)^(0) = 1 – *α* + *β*. Our measurement and fittings yield *g*^(2)^(0) = 0.22 for emitter SPE-1 and 0.01 for emitter SPE-2 without background correction, indicative of single photon emission (Figure 3c). Their excited-state lifetimes, as extracted from the *g*^(2)^(*t*) fitting, are 2.21 ns (SPE-1) and 2.71 ns (SPE-2), consistent with the typically reported excited-state lifetimes of SiV^−^ centers at room temperature [31, 49, 50]. The power-dependent emission intensities of SPEs are also recorded, revealing a typical saturated brightness of ∼70 kcps (Figure 3d). Note that the fluorescence was collected with an air objective configuration (Figure 3d inset). The brightness could be dramatically improved by employing an immersion oil objective with higher collection efficiency.

**Figure 4:**
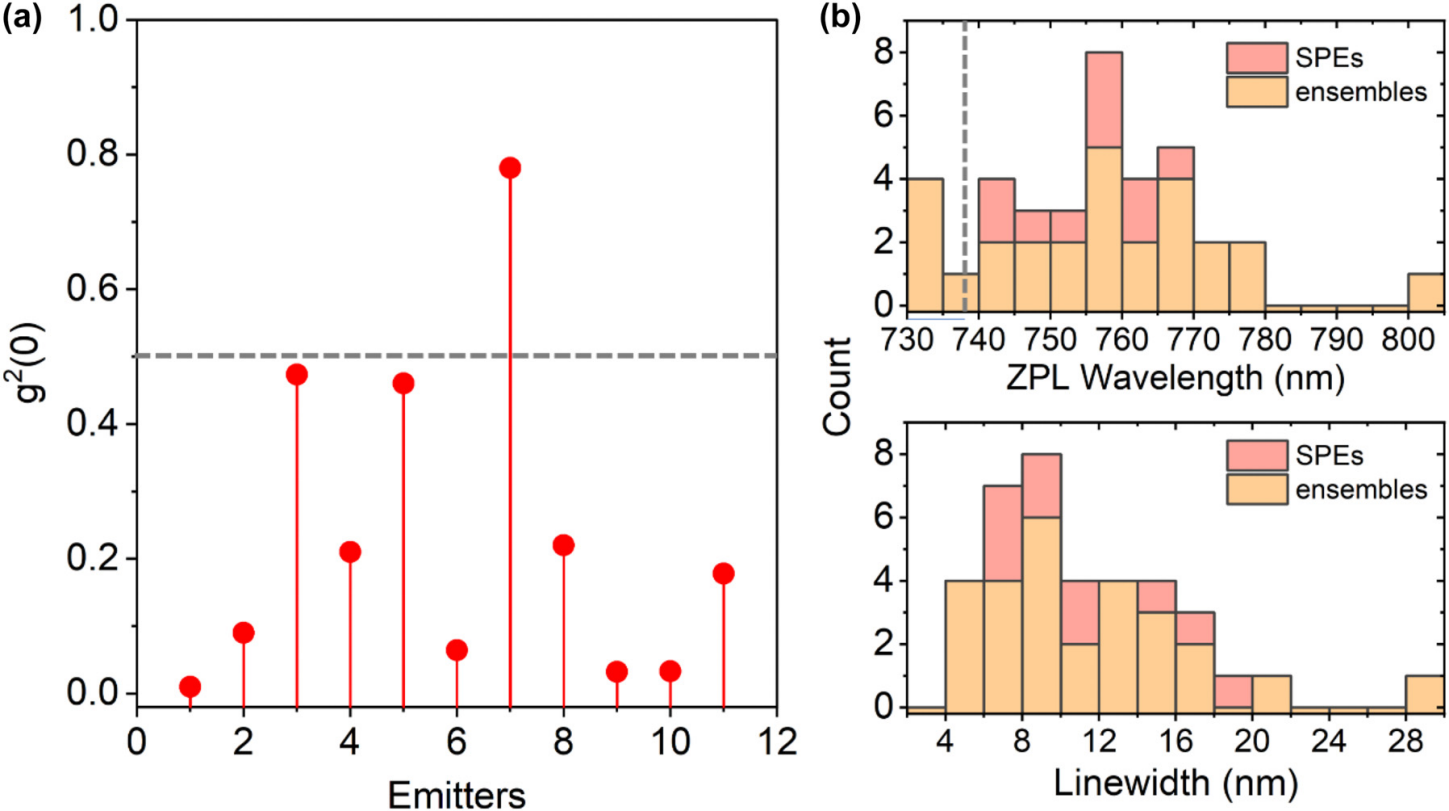
Statistics on *g*^2^(0), ZPL wavelength and linewidth of quantum emitters on sample D. (a) Distribution of Ga) Distribution of *g*^2^(0) values of 11 emitters found on sample D that show antibunched emission (*g*^2^(0) < 1). (b) Histograms of ZPL wavelengths (top) and linewidths (bottom) of 37 emitters from sample D. The data include both SPEs (red) and emitter ensembles (yellow). As a reference, the dashed gray line in the top panel of (b) indicates the typical emission wavelength of SiV^−^ centers in bulk diamond at room temperature (738 nm).

Aside from SPEs, we also notice emitters that show some antibunching but with *g*^2^(0) > 0.5. They are either composed of multiple SiV^−^ centers or are single defects accompanied by a strong fluorescence background. Figure 4a presents the *g*^2^(0) values of 11 emitters found on sample D featuring antibunched emission. Half of these emitters exhibit high single-photon purity with *g*^2^(0) below 0.1, without background correction.

The ZPL wavelengths and linewidths of all identified emitters vary significantly. We collected spectra of 37 stable emitters, including both SPEs and ensembles, and summarized the distribution of their linewidths and ZPL wavelengths (Figure 4b). Surprisingly, the ZPL wavelengths span a broad spectral range from 730 nm up to 803 nm, with the average wavelength being 757.0 nm (standard deviation 14.9 nm). The ZPL linewidth of emitters is measured in the range of 4 nm and 29 nm, with an average value of 11.2 nm (standard deviation 5.1 nm). Over 85% of emitters show a linewidth below 15 nm, similarly to previous reports on room-temperature SiV^−^ centers in NDs [45, 46].

## 3 Discussion

SiV^−^ centers in bulk diamond typically exhibit a ZPL at the wavelength of about 738 nm. In nanodiamonds, however, it is common to observe a wide distribution of both ZPL wavelengths and linewidths. Neu et al. [33, 48] synthesized NDs with sizes ranging from 75 to 160 nm using microwave plasma CVD and measured SiV centers emitting in the wavelength range of 730–750 nm. Lindner et al. [45] reported an even broader ZPL distribution of SiV^−^ centers (715–835 nm), with linewidths ranging from 1.4 nm to 18 nm, in NDs produced from wet milling of a CVD-grown diamond film. In the same study, they also reported ensembles of SiV^−^ centers in 250 nm-sized NDs, activated by high-energy ion implantation, that emit over a similarly broad spectral range as observed in this work. An important cause of the emission line shift and linewidth broadening is a higher strain or residual stress in diamond nanostructures compared to bulk diamond [48]. However, as Lindner et al. [45] pointed out, the stress-induced red shift of SiV^−^ ZPL should be within ∼10 nm as limited by the maximal stress in the diamond lattice. As a result, the broad distribution of ZPL wavelengths observed in our study cannot be solely attributed to strain. On the other hand, we observe no clear correlation between ZPL wavelengths and linewidths, suggesting that even the strongly red-shifted defects possess a similar atomic symmetry to the SiV^−^. Further investigations are needed to determine if the significant line shift results from other mechanisms or the formation of more complicated Si-related defects other than SiV^−^ centers. For example, measurements based on cryogenic photoluminescence excitation spectroscopy can provide us better insights into the photophysics of such emitters and confirm the atomic structure of such emitters. Having them in the form of singles hence provides an excellent platform for future research on this topic, free from the effects of center-center interaction and ensemble averaging.

The annealing procedure adopted in this work is first proposed by Takashima et al. [46] for creating ensembles of SiV^−^ centers in NDs, with emission linewidths as narrow as 6 nm. However, no clear evidence of single SiV^−^ center formation was found in their work, possibly due to unoptimized implantation energy and ion fluence. The formation of vacancy-related defects in a solid material depends on both the ion implantation energy and ion dosage. An increase in either ion energy or fluence results in more significant damage to the material [51, 52], hence increasing the vacancy concentration. In ref [46], ion energies starting at 30 keV were used, which was about two times higher than the ion energy used here. The more significant ion-induced damage at higher energies explains why SiV^−^ centers start to be observed at lower fluences (10^13^ ions/cm^2^) in their work. Nevertheless, such an ion energy-fluence combination might still have induced a high average concentration of SiV^−^ defects, favoring the formation of SiV^−^ ensembles. More details on the comparison of ref [46] and our work could be found in the Supplementary Materials.

Our experimental data allow us to estimate the yield of single color centers, i.e., the fraction of ND particles containing single emitters after ion implantation, on sample D: assuming a single layer of closely packed NDs with a size of 22.4 nm, we estimate the yield to be ∼(1 ± 0.5) × 10^−3^%. The actual value is likely higher since (i) ND particles are typically plate shaped with lateral sizes larger than heights (22.4 nm) and (ii) NDs might not be tightly packed in some areas – both factors lead to a smaller total number of NDs per unit area. Nevertheless, the yield is currently limited by the stochastic nature of emitter formation during ion implantation. Several strategies have been proposed to improve the yield of ion implantation-induced single emitters, potentially enabling the on-demand creation of single emitters in nanodiamonds. One strategy is the repeated low-dose ion implantation as proposed by Schröder et al. [53]. It was suggested that by repeating the “implantation – optical characterization – implantation” procedure, in combination with of the site-specific implantation capability of a focused ion beam, the chance of obtaining a certain number of emitters in a specific nanodiamond could be substantially improved. The uncertainty in emitter formation can also be reduced by controlling the number of implanted ions using *in situ* ion counting techniques [54, 55]. In combination with strategies to facilitate vacancy formation (e.g., laser or electron radiation [56, 57], coimplantation [58]), approaches mentioned above could potentially lead to the deterministic creation of single emitters in nanodiamonds. Aside from Si-related defects, the ion implantation technique can be expanded to the generation of other group-IV color centers in nanodiamonds, such as tin-vacancy and lead-vacancy centers, which so far are mostly limited to the bulk diamond platform [38, 59, 60].

Our work greatly expands the application of ion implantation in the field of quantum photonics. It opens up new possibilities to leverage NDs containing single narrowband color centers for quantum information technologies [61]. Color centers in sub-50 nm NDs are uniquely compatible with nanoscale plasmonic cavities [62] and hybrid plasmonic-dielectric resonators [63] that can provide orders of magnitude higher Purcell factors than dielectric resonators over orders of magnitude larger bandwidths. A previous report [64] has demonstrated coupling NDs containing color centers to plasmonic cavities with volumes as small as the NDs themselves and with mostly in-plane emission, compatible with on-chip integration. Integrating ND-based group IV color centers with nanophotonic structures promises ultrafast single photon emission of on-demand indistinguishable photons at cryo-free temperatures [63, 65]. The next essential step toward this goal is to create single SiV^−^ centers in isolated NDs with designated sizes, so diamond nanoparticles possessing suitable optical properties could be precisely identified and manipulated for plasmonic/photonic coupling. As mentioned previously, a focused ion beam [53] can be an essential tool to study and control the conditions for creating single SiV^−^ centers in isolated NDs. Last but not least, considering the biocompatibility of NDs and the SiV^−^ emission wavelength in the near-infrared regime, NDs with ion implantation-induced single SiV centers will contribute to the advancement of quantum-based bioimaging [66–68] at the nanoscale.

## 4 Materials and methods

### 4.1 ND sample preparation and preliminary characterization

Nonfluorescent NDs (Adamas Nanotechnologies) with an average size of 20–30 nm containing no color centers were suspended in DI water (4 mg/mL). The NDs were synthesized via the high-pressure high-temperature (HPHT) method and not previously doped. The original suspension is diluted with DI water to 400 μg/mL, sonicated for ∼60 min, drop casted onto round 1-inch quartz coverslips (SPI supplies), and dried in vacuum. Before drop casting, the coverslips were rinsed with acetone, methanol, and isopropanol and placed in an ozone cleaner for 60 min. Finally, the samples are rinsed with DI water to remove contaminants.

Before implanting Si into the NDs, we prepared two control samples. The first control sample with sparse NDs on a coverslip glass (Fisher Scientific) served to characterize the ND size distribution using an atomic force microscope (Cypher S AFM, Asylum Research). It was prepared using the above procedure with a more diluted suspension (4 μg/mL). A second control sample was made to examine the crystalline structure of NDs using a vacuum transmission electron microscope (TEM) (FEI Themis Z). It was prepared by drop casting the ND solution onto a carbon membrane-based TEM grid (Ted Pella) and drying it in vacuum. An acceleration voltage of 200 kV was used in the TEM mode characterization to reduce electron damage to NDs.

### 4.2 Ion implantation and annealing

All the ND samples on quartz substrate were implanted with a large-area silicon (^28^Si) ion implanter (200 kV Varian Ion Implant System at Los Alamos National Laboratory) by irradiating the entire sample surface. An ion energy of 12 kV was utilized, corresponding to an estimated implantation depth of 10 ± 3 nm according to the Stopping and Range of Ions in Matter (SRIM) simulation (Figure 2a). Four implantation fluences at room temperature, 2 × 10^10^, 1 × 10^12^, 5 × 10^13^, and 1 × 10^15^ ions/cm^2^, were tested and noted as sample A, B, C, and D, respectively. After ion implantation, the samples were annealed according to the procedure reported in ref [46]. Typical ultra-high vacuum anneals used for color center activation in bulk diamond showed no success in activating SiV^−^ in NDs [55]. The annealing process was not only essential for activating and stabilizing color centers but also helped to remove amorphous carbon on ND surface induced by ion implantation.

### 4.3 Optical characterization

We performed optical characterization of implanted SiV^−^ centers at room temperature using a custom-made scanning confocal microscope based on a commercial inverted microscope body (Ti-U, Nikon). The setup has a collection efficiency of ∼20%, without considering the objective collection efficiency. An air objective with a numerical aperture of 0.90 was used to excite the color centers and collect the photoemission. The excitation light and PL signal were separated by a 700 nm long-pass dichroic mirror (FF700-Di01-50.8-D 700 nm BrightLine long-pass filter, Semrock). The collected light was then spatially filtered by a 100-μm pinhole, and the remaining pump beam was filtered out by a 715 nm long-pass filter (FF01-715/LP-25 715 nm BrightLine long-pass filter, Semrock) installed in front of the detectors. After passing through the pinhole, two avalanche photodetectors with 30-ps time resolution and 35% quantum efficiency at 650 nm (PDM, Micro-Photon Devices) were used for single-photon detection during lifetime and autocorrelation measurements. An avalanche detector with 69% quantum efficiency at 650 nm (SPCM-AQRH, Excelitas) was used for scanning and saturation measurements. We used a continuous-wave 690 nm diode-pumped solid-state laser (DL690-050, CrystaLaser) for the optical excitation of emitters. A 700 nm short-pass filter was installed in front of the laser to remove any light coming from incoherent long wavelength emission from the laser cavity (FES0700, Thorlabs). For lifetime measurements, we used a dispersion-compensated Ti:Sapphire mode-locked laser (Mai Tai DeepSee, Spectra-Physics) operating at 690 nm with a nominal 80 MHz repetition rate and a pulse duration of about 200 fs at the microscope sample plane.

## Supplementary Material

Supplementary Material Details
