# Supplementary material for: Fabrication of single color centers in sub-50 nm nanodiamonds using ion implantation
Source: Nanophotonics. 2023 Feb 1;12(3):485–94. doi: 10.1515/nanoph-2022-0678 (PMC11501155; doi:10.1515/nanoph-2022-0678)
Supplement: Supplementary file 1 — Supplementary Material Details [file j_nanoph-2022-0678_suppl.pdf]

Supplementary Materials

Xiaohui Xu, Zachariah O. Martin, Michael Titze, Yongqiang Wang, Demid Sychev, Jacob Henshaw, Alexei S. Lagutchev, Han Htoon, Edward S. Bielejec, Simeon I. Bogdanov, Vladimir M. Shalaev, Alexandra Boltasseva*

Fabrication of Single Color Centers in Sub-50 nm Nanodiamonds Using Ion Implantation

1. Characterization of nanodiamonds after ion implantation and annealing

The size of nanodiamonds (NDs) could change due to the damage induced by ion implantation and annealing, so it is essential to check NDs with AFM after the implantation/annealing procedure. Given that all ND samples were implanted under the same ion energy and annealed with the same procedure, we started with sample D implanted with the highest ion fluence (1x10^{15} ions/cm^2), which is expected to be impacted the most by implantation/annealing. Since sample D was prepared from a high-concentration ND dispersion, most of the sample area contains densely packed NDs that are not suitable for particle size/height measurement, as the particles might stack up along the thickness direction. We were able to find areas near the outer edge of the sample surface with sparse diamond particles (Figure S1a), which provides us better insights into the height/size information of NDs. Figure S1b shows the size distribution of 412 particles from the area in Figure S1a, ranging from 12 nm to 34 nm. An average size of 19.1 ± 5.3 nm was obtained, slightly smaller than the average size measured for NDs before implantation and annealing (22.4 ± 6.9 nm). Considering that the measured size difference is only ~ 3 nm, it could be caused by either the particle size variation in the original ND dispersion or the ion implantation/annealing effect. Nevertheless, our

*Corresponding author: Alexandra Boltasseva, Elmore Family School of Electrical and Computer Engineering, School of Materials Engineering, Birck Nanotechnology Center, Purdue Quantum Science and Engineering Institute (PQSEI), Purdue University, West Lafayette, Indiana 47907, USA, The Quantum Science Center (QSC), a National Quantum Information Science Research Center of the U.S. Department of Energy (DOE), Oak Ridge National Laboratory, Oak Ridge, TN 37831, USA. Email: aeb@purdue.edu.
Xiaohui Xu, School of Materials Engineering, Birck Nanotechnology Center, Purdue University, West Lafayette, Indiana 47907, USA.
Zachariah O. Martin, Demid Sychev, Alexei S. Lagutchev, Elmore Family School of Electrical and Computer Engineering, Birck Nanotechnology Center, Purdue University, West Lafayette, Indiana 47907, USA.
Vladimir M. Shalaev, Elmore Family School of Electrical and Computer Engineering, Birck Nanotechnology Center, Purdue Quantum Science and Engineering Institute (PQSEI), Purdue University, West Lafayette, Indiana 47907, USA, The Quantum Science Center (QSC), a National Quantum Information Science Research Center of the U.S. Department of Energy (DOE), Oak Ridge National Laboratory, Oak Ridge, TN 37831, USA.
Michael Titze, Jacob Henshaw, Edward S. Bielejec, Sandia National Laboratories, Albuquerque, New Mexico 87123, USA.
Yongqiang Wang, Han Htoon, Los Alamos National Laboratory, Los Alamos, 87545, New Mexico, USA
Simeon I. Bogdanov, Department of Electrical and Computer Engineering, Nick Holonyak, Jr. Micro and Nanotechnology Laboratory, Illinois Quantum Information Science and Technology Center, University of Illinois at Urbana-Champaign, Urbana, Illinois, 60801, USA.

measurements confirm that the ion implantation/annealing procedure has minimal effects on the size of NDs.

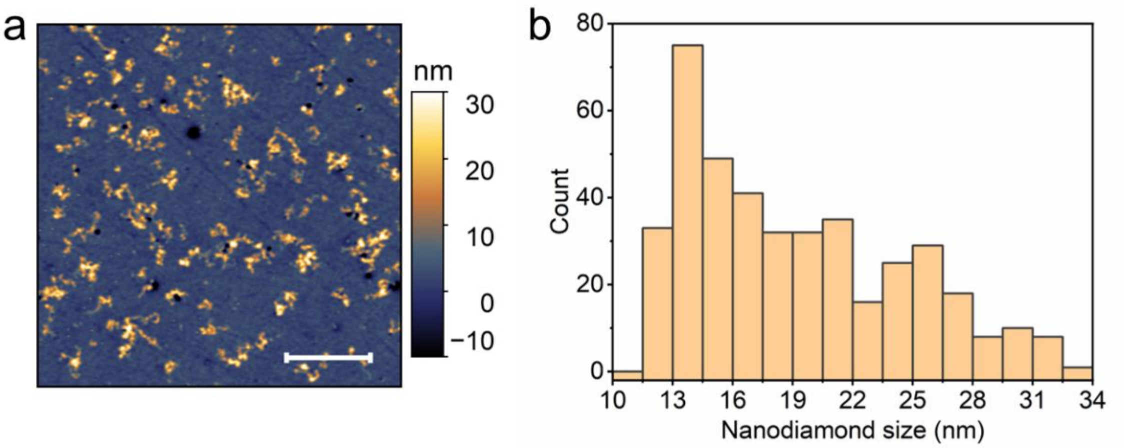

Figure S1. *(a) AFM image of an area containing NDs from sample D (implanted at 1 x 10^{15} ions/cm^2). In contrast to the area shown in Figure 1d of the main paper, this area contains much sparser NDs as it was located at the outer edge of the sample area where NDs were drop-casted. Scale bar: 4 µm. (b) Histogram showing the ND size distribution. Sizes are collected from 412 NDs recognized from the AFM scan in (a). Particles with heights < 12 nm are excluded from the statistics as they could be dirt or features on the substrates.*

2. Comparison of our work with ref[46] in the main text

In the work by H. Takashima *et al* (ref[46] in the main text), the authors reported the creation of ensembles of silicon vacancy color center in nanodiamonds using the ion implantation + annealing approach. Sharp SiV^- emission was observed at the ion fluence of 10^{13} ions/cm^2 under the ion energy of 30 keV and 180 keV. Further increasing the ion fluence to 2 x 10^{15} ions/cm^2 leads to the broadening and red shift of the SiV^- ZPL. This is in contrast with our observation where sharp SiV^- emission starts to appear at an ion fluence as high as 1 x 10^{15} ions/cm^2 under an ion energy of 12 keV. The difference above could be explained by the following reasons:

1) NDs with different sizes were used, i.e., ~ 22 nm in our study and ~ 30 nm by H. Takashima *et al* for the smallest NDs they tested. As a result, different implantation energies had to be used to achieve desired ion penetration depths. The ion energy of 30 keV used by H. Takashima *et al* corresponds to an ion penetration depth of 22 nm, which cannot be applied to our study considering the size of our NDs.
2) The number of SiV^- centers is a nonlinear function of the ion energy and dose since both factors affect the concentration and distribution of vacancies in diamond, which in turn affects the probability that a Si atom captures a vacancy for SiV^- formation. This effect has been demonstrated before, for example, by T. Schroder *et al* [1]. As a result, the optimal dose reported in Reference 46 is not necessarily the optimal dose for our study.

3) Substrates could also play a role. Instead of using silicon substrates as H. Takashima *et al* did, we used quartz substrates that show stronger charging effect, which could modify the effective ion fluences on the NDs, leading to an optimal ion fluence that is different from the previous report.

References

[1] T. Schröder et al., *Scalable Focused Ion Beam Creation of Nearly Lifetime-Limited Single Quantum Emitters in Diamond Nanostructures*, Nat. Commun. **8**, (2017).
